# Identification of Risk Factors Influencing Hemorrhage Volume in Aneurysmal Subarachnoid Hemorrhage: A Multicenter Retrospective Study

**DOI:** 10.1002/brb3.70498

**Published:** 2025-05-08

**Authors:** Chenglong Li, Yuan Wang, Tangming Peng, Jiangnan Wu, Hongyu Wang, Jian Song, Di Zhao, Guang Feng, Lei Chen

**Affiliations:** ^1^ Department of Neurosurgery Shanxi Provincial People's Hospital Taiyuan Shaanxi China; ^2^ Department of Neurosurgery Tangshan Gongren Hospital Tangshan Hebei China; ^3^ Department of Neurosurgery, Chengdu Fifth People's Hospital The Fifth People's Hospital Affiliated to Chengdu University of Traditional Chinese Medicine Chengdu China; ^4^ Department of Artificial Intelligence Tianjin University of Technology Tianjin China; ^5^ Department of Neurosurgery The Second Hospital of Hebei Medical University Shijiazhuang Hebei China; ^6^ Department of Imaging The Second Hospital of Hebei Medical University Shijiazhuang Hebei China; ^7^ The Neurosurgical Intensive Care Unit Henan Provincial People's Hospital Zhengzhou Henan China; ^8^ Department of Neurosurgery The Fourth Hospital of Hebei Medical University Shijiazhuang Hebei China

**Keywords:** aneurysm morphology, aneurysmal subarachnoid hemorrhage, diabetes, hemorrhage volume, hypertension, risk factors

## Abstract

**Objective:**

This multicenter retrospective study aimed to identify significant risk factors influencing hemorrhage volume in patients with aneurysmal subarachnoid hemorrhage (SAH).

**Methods:**

A total of 891 patients diagnosed with SAH were included from multiple medical centers. Data encompassing demographic characteristics, medical history, clinical parameters at admission, and radiographic findings were collected and analyzed. Univariate and multivariate logistic regression analyses were conducted to investigate associations between various risk factors and hemorrhage volume.

**Results:**

This study identifies several factors significantly associated with increased hemorrhage volume in patients with subarachnoid hemorrhage (SAH). Multivariate analysis revealed that diabetes (P = 0.022), hypertension (P = 0.047), and saccular aneurysm morphology (P = 0.008) were independent risk factors for high hemorrhage volume. Additionally, larger aneurysm size (maximum diameter: P = 0.007, neck diameter: P = 0.021) and higher systolic blood pressure after onset (P = 0.002) were also significant predictors of increased hemorrhage volume. Factors such as age (P = 0.05) and time interval to the first CT scan (P = 0.022) were found to be associated with hemorrhage volume in univariate analysis but did not maintain independent significance in multivariate regression.

**Conclusion:**

This study highlights key risk factors, including diabetes, hypertension, and saccular aneurysm morphology, which independently contribute to higher hemorrhage volume in SAH patients. Management strategies focusing on early detection and control of these factors may improve clinical outcomes by reducing the risk of hemorrhagic complications. While other factors such as age and time interval to the first CT scan were associated with hemorrhage volume, they did not demonstrate independent causality in the multivariate analysis, suggesting that their role in hemorrhage volume may be secondary or context‐dependent.

## Graphical abstract

Early identification and management of some risk factors can improve clinical outcomes and reduce the risk of bleeding complications.

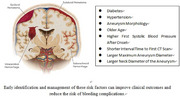

## 1 Introduction

Aneurysmal subarachnoid hemorrhage (aSAH) is an acute cerebrovascular disease characterized by the rupture of an intracranial aneurysm, resulting in the leakage of blood into the subarachnoid space (Suarez et al. 2006; Neifert et al. 2021). This condition accounts for approximately 85% of all subarachnoid hemorrhage (SAH) cases and has an incidence rate of 6–9 cases per 100,000 individuals per year (Terpolilli et al. 2015). The mortality rate is alarmingly high, with 30–50% of patients succumbing within 30 days of onset (Bolouki et al. 2019). Furthermore, about half of the survivors experience permanent neurological deficits, significantly impacting their quality of life and imposing a substantial socioeconomic burden (Terpolilli et al. 2015; Ru et al. 2021).

The volume of hemorrhage is a crucial determinant of prognosis in SAH patients. Extensive bleeding can lead to severe complications such as elevated intracranial pressure, cerebral vasospasm, and delayed ischemic neurological deficits, markedly increasing the risk of mortality and morbidity (Wen et al. 2022; Kramer et al. 2021; Akgun and Akgul 2023). Therefore, understanding the risk factors influencing hemorrhage volume is of paramount clinical importance.

Advancements in neuroimaging, particularly computed tomography (CT) and magnetic resonance imaging (MRI), have greatly enhanced the early diagnosis and precise assessment of hemorrhage volume in SAH patients (Autio et al. 2023; Ryan and Jaju 2023). While these technological advances primarily support diagnosis, they cannot directly influence prognosis or outcomes. However, they indirectly inform more precise therapeutic approaches, which in turn may improve patient outcomes. Despite these advances, the prognosis for SAH patients remains suboptimal, emphasizing the need for improved prevention and early intervention strategies. These strategies rely on a comprehensive understanding of the risk factors that influence hemorrhage volume.

Previous studies have identified several risk factors associated with SAH, including hypertension, smoking, excessive alcohol consumption, and family history (Ellamushi et al. 2001; Kubota et al. 2001; Tozawa et al. 2000). However, findings regarding the specific factors affecting hemorrhage volume have been inconsistent, often derived from single‐center studies with small sample sizes. For instance, some studies suggest that age and gender may impact hemorrhage volume, yet these results are contentious (Kubota et al. 2001; Tozawa et al. 2000; Jabbarli et al. 2016). Additionally, critical factors such as aneurysm morphology and underlying medical conditions have not been thoroughly investigated.

With the demographic shifts towards an aging population and changes in lifestyle, the epidemiology of SAH is evolving. For example, the rising prevalence of diabetes may influence the incidence and severity of SAH (Jardim et al. 2014). Consequently, it is essential to reassess and update the risk factors affecting hemorrhage volume in the context of these new societal dynamics.

This study aims to identify and analyze key factors influencing hemorrhage volume in SAH patients through a comprehensive, multicenter, retrospective data analysis. By evaluating a wide range of potential risk factors, we seek to develop more accurate predictive tools for clinical practice, aiding healthcare professionals in the early identification of high‐risk patients and the formulation of personalized treatment strategies.

## 2 Materials and Methods

### 2.1 Study Design and Participants

This study conducted a multicenter retrospective analysis to identify risk factors influencing hemorrhage volume in patients diagnosed with aneurysmal subarachnoid hemorrhage (SAH). The study included a total of 891 patients recruited from multiple medical centers by The Fourth Hospital of Hebei Medical University, with participation from The Second Hospital of Hebei Medical University, Tangshan Workers' Hospital, Henan Provincial People's Hospital, Shanxi Provincial People's Hospital, and the Affiliated Hospital of Southwest Medical University. Patients with confirmed subarachnoid hemorrhage (SAH) via CT and diagnosed with intracranial aneurysm through CTA or DSA were included in this study. This study was conducted in accordance with the principles of the Declaration of Helsinki and approved by the institutional review boards of all participating centers. Informed consent was waived due to the retrospective nature of the study and anonymization of patient data.

### 2.2 Data Collection

Data were collected retrospectively from the participating centers from 2023.12‐2024.06. Information retrieved included demographic details (age, gender), clinical characteristics (hypertension, diabetes, smoking status, alcohol consumption), laboratory findings (hemoglobin level, hematocrit level, platelet count, coagulation parameters), aneurysm characteristics (maximum diameter, neck diameter, morphology), and clinical outcomes (hemorrhage volume categorized by modified Fisher scale (Frontera et al. 2006, Páez‐Granda et al. 2021)). Grades 0, I, and II were classified as the low hemorrhage volume (low volume) group, while grades III and IV were classified as the high hemorrhage volume (high volume) group.

### 2.3 Risk Factors Assessed

#### 2.3.1 The Study Included a Comprehensive Set of Potential Risk Factors

##### 2.3.1.1 Demographic Information

Gender, age, history of hypertension, diabetes, hyperlipidemia, smoking history, alcohol consumption history, allergy history, family history of cerebrovascular disease, and family history of aneurysm disease.

##### 2.3.1.2 Disease‐Related Risk Factors

History of previous aneurysm rupture, post‐onset blood pressure values (systolic and diastolic), time interval to the first CT scan, occurrence of secondary hemorrhage events, emergency examination results indicating elevated blood lipids, elevated uric acid, elevated homocysteine, hemoglobin values, hematocrit values, platelet count, coagulation function values (PT, APTT, TT, INR, FIB), aneurysm morphology, number of aneurysms, location of the responsible aneurysm, and related data of the responsible aneurysm (maximum diameter, neck diameter, aneurysm‐to‐neck ratio).

### 2.4 Statistical Analysis

Statistical analyses were performed using appropriate software (e.g., SPSS, R). Descriptive statistics were used to summarize demographic, clinical, and laboratory characteristics of the study population. Categorical variables were presented as frequencies and percentages, while continuous variables were expressed as means with standard deviations. Univariate analyses were conducted using chi‐square tests or independent t‐tests to assess associations between categorical and continuous variables, respectively, and hemorrhage volume. Variables demonstrating significance (P < 0.1) in univariate analysis were further evaluated in a multivariate logistic regression model to identify independent risk factors for high hemorrhage volume.

## 3 Results

### 3.1 Patient Demographics and Clinical Characteristics

A total of 891 patients with aneurysmal subarachnoid hemorrhage (SAH) were included in this multicenter retrospective study. The majority of patients were female (62.7%) and had a history of hypertension (49.8%). Other prevalent conditions included smoking (22.9%), alcohol consumption (16.7%), and diabetes (8.2%). The modified Fisher scale categorized 65.7% of patients into the low hemorrhage volume group (grades 0, I, II), and 34.3% into the high hemorrhage volume group (grades III, IV). As shown in Table 1.

**TABLE 1:** Patient demographics and clinical characteristics.

Category	Option	Frequency	Percentage (%)
Gender	Female	559	62.738
	Male	332	37.262
Hypertension	No	444	49.832
	Yes	444	49.832
	Unknown	3	0.337
Diabetes	No	814	91.358
	Yes	73	8.193
	Unknown	4	0.449
Hyperlipidemia	No	741	83.165
	Yes	23	2.581
	Unknown	127	14.254
Smoking	No	680	76.319
	Yes	204	22.896
	Unknown	7	0.786
Alcohol consumption	No	739	82.941
	Yes	149	16.723
	Unknown	3	0.337
Drug or food allergy	No	879	98.653
	Yes	10	1.122
	Unknown	2	0.224
History of aneurysmal subarachnoid hemorrhage	No	856	96.072
	Yes	35	3.928
Family history of cerebrovascular disease	No	859	96.409
	Yes	17	1.908
	Unknown	15	1.684
Medication intervention for hypertension	No	455	51.066
	Yes	345	38.721
	Unknown	91	10.213
Preoperative rebleeding	Yes	44	4.938
	No	847	95.062
Elevated blood lipids	No	296	33.221
	Yes	154	17.284
	Unknown	441	49.495
Elevated uric acid	No	627	70.37
	Yes	51	5.724
	Unknown	213	23.906
Aneurysm morphology	Saccular aneurysm	693	77.778
	Fusiform aneurysm	76	8.53
	Dissecting aneurysm	52	5.836
	Mycotic aneurysm	35	3.928
	False aneurysm	19	2.132
	Blood blister aneurysm	16	1.796
Number of aneurysms	1	701	78.676
	2	151	16.947
	3	24	2.694
	> 3	15	1.684
Aneurysm location	Anterior circulation aneurysm	794	89.113
	Posterior circulation aneurysm	97	10.887
Modified fisher scale	Low volume	585	65.657
	High volume	306	34.343

The mean age was 57.23 years. Initial systolic blood pressure averaged 141.407 mmHg, and diastolic blood pressure averaged 83.762 mmHg. The mean time interval to the first CT scan was 23.903 min. Hematologic parameters revealed a mean hemoglobin level of 131.063 g/L, hematocrit level of 35.237%, and platelet count of 227.472 × 10^9/L. Coagulation function assessments showed mean prothrombin time (PT) of 12.316 seconds, international normalized ratio (INR) of 1.294, activated partial thromboplastin time (APTT) of 28.367 seconds, and thrombin time (TT) of 15.835 seconds. Plasma fibrinogen (Fib) level averaged 3.237 g/L. Aneurysm characteristics indicated a mean maximum diameter of 5.184 mm and mean neck diameter of 3.514 mm. As shown in Table 2.

**TABLE 2:** Patient Laboratory and Clinical Data.

Variable name	Sample size	Maximum value	Minimum value	Mean value	Standard deviation
Age	888	86	3	57.23	12.167
First recorded systolic blood pressure	891	239	75	141.407	22.497
First recorded diastolic blood pressure	891	160	40	83.762	13.715
Time to first CT scan (minutes)	890	720	0	23.903	53.279
Hemoglobin (Hb)	889	189	3.85	131.063	20.257
Hematocrit (Hct)	889	445	0.15	35.237	23.153
Platelets (Plt)	889	870	3.5	227.472	72.594
Prothrombin time (PT)	880	117	8.9	12.316	5.97
International normalized ratio (INR)	880	124	0.72	1.294	5.56
Activated partial thromboplastin time (APTT)	880	67	0.89	28.367	4.753
Thrombin time (TT)	852	240	2.54	15.835	10.205
Plasma fibrinogen (Fib)	873	40.75	0.49	3.237	1.538
Maximum aneurysm diameter	871	40	0.3	5.184	3.741
Aneurysm neck diameter	865	26	0.2	3.514	2.28

### 3.2 Univariate Analysis of Risk Factors Associated With Hemorrhage Volume

Categorical variables analyzed included gender, hypertension, diabetes, hyperlipidemia, smoking, alcohol use, drug or food allergies, history of aneurysmal subarachnoid hemorrhage, family history of cerebrovascular disease, drug intervention for hypertension, and preoperative rebleeding. Among these, hypertension (χ^2^ = 4.488, P = 0.034), diabetes (χ^2^ = 6.365, P = 0.012), and aneurysm morphology (χ^2^ = 16.349, P = 0.006) showed significant associations with high hemorrhage volume. Specifically, patients with hypertension and diabetes were more likely to have high hemorrhage volume, while saccular aneurysms demonstrated a higher prevalence in the high volume group compared to other morphologies.

Continuous variables analyzed included age (T = ‐2.372, P = 0.018), first systolic blood pressure (refers to the initial systolic blood pressure measurement taken after the clinical onset of the disease, typically within the first few hours following the onset of symptoms). This value serves as an early indicator of the patient's hemodynamic status and may reflect the severity of the condition after onset (T = ‐3.057, P = 0.002), time interval to the first CT scan (T = 2.29, P = 0.022), maximum diameter of the aneurysm (T = ‐3.409, P = 0.001), and neck diameter of the aneurysm (T = ‐2.306, P = 0.021). These findings indicate that older age, higher systolic blood pressure after onset, shorter time interval to the first CT scan, larger maximum aneurysm diameter, and larger neck diameter are associated with increased hemorrhage volume in this patient cohort.

Variables where the differences did not reach statistical significance include hyperlipidemia, smoking, alcohol use, drug or food allergies, history of aneurysmal subarachnoid hemorrhage, family history of cerebrovascular disease, drug intervention for hypertension, preoperative rebleeding, hyperuricemia, and various coagulation parameters. As shown in Table 3.

**TABLE 3:** Univariate analysis of differences between low and high hemorrhage volume groups.

		Improved fisher classification				
Topic	Name	Low volume	High volume	Total	χ^2^/t	P
Gender				891	χ^2^ = 0.344	0.557
	Female	363	196	559		
	Male	222	110	332		
Hypertension				888	χ^2^ = 4.488	0.034**
	No	306	138	444		
	Yes	276	168	444		
Diabetes				887	χ^2^ = 6.365	0.012**
	No	543	271	814		
	Yes	38	35	73		
Hyperlipidemia				764	χ^2^ = 2.653	0.103
	No	506	235	741		
	Yes	12	11	23		
Smoking				884	χ^2^ = 1.676	0.195
	No	440	240	680		
	Yes	142	62	204		
Alcohol				888	χ^2^ = 0.523	0.47
	No	489	250	739		
	Yes	94	55	149		
Drug, food allergy				889	χ^2^ = 0.002	0.963
	No	578	301	879		
	Yes	6	4	10		
History of aneurysmal subarachnoid hemorrhage				891	χ^2^ = 3.271	0.071*
	No	567	289	856		
	Yes	18	17	35		
Family history of cerebrovascular disease				876	χ^2^ = 0.18	0.671
	No	564	295	859		
	Yes	12	5	17		
Drug intervention in patients with hypertension				800	χ^2^ = 1.718	0.19
	No	323	132	455		
	Yes	230	115	345		
Preoperative rebleeding				891	χ^2^ = 0.001	0.971
	No	556	291	847		
	Yes	29	15	44		
Hyperlipidemia				450	χ^2^ = 0.21	0.647
	No	191	105	296		
	Yes	96	58	154		
Hyperuricemia				678	χ^2^ = 0.419	0.517
	No	402	225	627		
	Yes	35	16	51		
Aneurysm morphology				891	χ^2^ = 16.349	0.006***
	Saccular Aneurysm	454	239	693		
	Lobulated Aneurysm	48	28	76		
	Blister‐like Aneurysm	5	11	16		
	Dissecting Aneurysm	21	14	35		
	Fusiform Aneurysm	43	9	52		
	Pseudoaneurysm	14	5	19		
Number of brain aneurysms				891	χ^2^ = 7.304	0.063*
	1	473	228	701		
	2	85	66	151		
	3	16	8	24		
	> 3	11	4	15		
Aneurysm location				891	χ^2^ = 0.146	0.702
	Anterior circulation aneurysm	523	271	794		
	Posterior circulation aneurysm	62	35	97		
Age		56.53±12.01	58.56±12.37	888	T = ‐2.372	P = 0.018**
First systolic blood pressure after onset		139.75±21.59	144.58±23.86	891	T = ‐3.057	P = 0.002***
First diastolic blood pressure after onset		83.15±13.82	84.93±13.47	891	T = ‐1.838	P = 0.066*
Interval time to first CT Scan		26.86±49.41	18.27±59.66	890	T = 2.29	P = 0.022**
Hemoglobin (Hb)		131.79±19.44	129.68±21.69	889	T = 1.481	P = 0.139
Hematocrit (Hct)		36.11±26.82	33.57±13.57	889	T=1.557	P = 0.120
Platelet (Plt)		227.73±71.31	226.98±75.10	889	T = 0.146	P = 0.884
Plasma prothrombin time (PT)		12.29±5.85	12.37±6.20	880	T = ‐0.195	P = 0.845
International normalized ratio (INR)		1.44±6.85	1.01±0.15	880	T = 1.107	P = 0.269
Activated partial thromboplastin time (APTT)		28.41±4.59	28.29±5.06	880	T = 0.353	P = 0.724
Thrombin time (TT)		15.59±10.63	16.31±9.34	852	T = ‐0.972	P = 0.331
Plasma fibrinogen (Fib)		3.17±0.81	3.37±2.37	873	T = ‐1.824	P = 0.069*
Maximum diameter of tumor body		4.88±3.39	5.78±4.30	871	T = ‐3.409	P = 0.001***
Neck diameter of tumor		3.39±2.20	3.76±2.41	865	T = ‐2.306	P = 0.021**

*Note*: ***, **, * represent p < 0.01, p < 0.05, p < 0.1 respectively.

### 3.3 Multivariate Analysis of Risk Factors for High Hemorrhage Volume

In the multivariate logistic regression analysis, factors independently associated with high hemorrhage volume included maximum diameter of the aneurysm (OR = 1.079, 95% CI 1.021‐1.141, P = 0.007), diabetes (OR = 1.819, 95% CI 1.089‐3.039, P = 0.022), time interval to the first CT scan (OR = 0.995, 95% CI 0.992‐0.999, P = 0.007), first systolic pressure after onset (OR = 1.007, 95% CI 1.000‐1.014, P = 0.047), and saccular aneurysm shape (OR = 9.005, 95% CI 1.764‐45.979, P = 0.008). Other variables such as age, aneurysm neck diameter, hypertension, and specific aneurysm shapes (lobulated, cystic, dissecting, fusiform) did not show significant independent associations with high hemorrhage volume (P > 0.05). As shown in Table 4.

**TABLE 4:** Multivariate analysis of risk factors for high hemorrhage volume in aneurysmal subarachnoid hemorrhage.

						95% Confidence interval	
	Regression coefficient	Standard error	Wald	P	OR	Upper limit	Lower limit
Constant	−2.984	0.808	13.642	0.000***	0.051	0.01	0.246
Age	0.009	0.006	1.97	0.16	1.009	0.996	1.022
First systolic pressure after onset	0.007	0.004	3.932	0.047**	1.007	1	1.014
Interval time from first CT scan	−0.005	0.002	7.294	0.007***	0.995	0.992	0.999
Maximum diameter of tumor body	0.076	0.028	7.204	0.007***	1.079	1.021	1.141
Diameter of tumor neck	0.002	0.045	0.001	0.97	1.002	0.917	1.094
Diabetes_yes	0.599	0.262	5.231	0.022**	1.819	1.089	3.039
Hypertension_yes	0.148	0.16	0.858	0.354	1.16	0.847	1.588
Aneurysm shape_lobulated aneurysm	0.472	0.594	0.633	0.426	1.604	0.501	5.133
Aneurysm shape_cystic aneurysm	0.378	0.543	0.484	0.487	1.459	0.503	4.234
Aneurysm shape_dissecting aneurysm	0.472	0.655	0.52	0.471	1.603	0.444	5.784
Aneurysm shape_fusiform aneurysm	∕0.537	0.654	0.673	0.412	0.585	0.162	2.108
Aneurysm shape_saccular aneurysm	2.198	0.832	6.98	0.008***	9.005	1.764	45.979

*Note*: ***, **, * represent p< 0.01, p< 0.05, p< 0.1 respectively.

## 4 Discussion

This multicenter retrospective study identifies several significant risk factors associated with high hemorrhage volume in patients with aneurysmal subarachnoid hemorrhage (aSAH). These findings enhance our understanding of the factors influencing hemorrhage volume and provide important insights for risk assessment and clinical decision‐making in practice.

Our study population exhibited a higher proportion of females (62.7%), consistent with previous reports on the gender distribution of aSAH (Rehman et al. 2020; Lindekleiv et al. 2011). The average age was 57.23 years, slightly lower than that reported in some developed countries (Nieuwkamp et al. 2009; Dabilgou et al. 2019), possibly reflecting regional demographic differences. Hypertension, observed in 49.8% of patients, was the most common comorbidity, which underscores the critical role of blood pressure management in the prevention and treatment of aSAH (Ramesh et al. 2020; Fan et al. 2022).

### 4.1 Factors Influencing Hemorrhage Volume

#### 4.1.1 Multivariate Analysis Revealed Several Independent Risk Factors for High Hemorrhage Volume

##### 4.1.1.1 Maximum Aneurysm Diameter

Each 1 mm increase in aneurysm diameter was associated with a 7.9% increase in the risk of high hemorrhage volume (OR = 1.079). This finding corroborates the results of Guo et al., emphasizing the need for close monitoring and aggressive intervention in patients with large aneurysms (Guo et al. 2011). As the diameter increases, the stress on the aneurysm wall also increases, leading to a higher risk of rupture. Larger aneurysms exhibit more complex internal blood flow patterns, which can result in turbulent flow and abnormal distribution of wall shear stress, further increasing the risk of damage to the aneurysm wall (Rostamian et al. 2023; Sadeh et al. 2023).

##### 4.1.1.2 Diabetes

The presence of diabetes increased the risk of high hemorrhage volume by 81.9% (OR = 1.819). This result is in line with Lindbohm's research (Lindbohm et al. 2016), suggesting that diabetes may exacerbate vascular fragility, highlighting the importance of glucose control in the management of aSAH patients. Diabetes may increase the volume of subarachnoid hemorrhage (SAH) through various mechanisms. These include endothelial dysfunction, making blood vessel walls more prone to rupture; coagulation abnormalities, making bleeding harder to control; chronic inflammation, weakening the vessel walls; hypertension, a common comorbidity and a major risk factor for hemorrhagic events; and oxidative stress and long‐term vascular damage caused by diabetes (Wang et al. 2008; Feng et al. 2022; Andreasen et al. 2013). These factors collectively make diabetic patients more susceptible to severe bleeding. While there are currently no direct studies specifically linking diabetes to increased SAH volume, these mechanisms provide a plausible explanation for why diabetes might exacerbate hemorrhage in SAH.

##### 4.1.1.3 Time Interval to First CT Scan

Interestingly, each minute delay in the time interval to the first CT scan was associated with a 0.5% (OR = 0.995) decrease in the risk of high hemorrhage volume, which may reflect the correlation between more severe cases and faster imaging. This does not imply that delaying imaging would reduce bleeding risk, but rather suggests that quicker imaging is typically seen in more severe cases. This seemingly paradoxical result may indicate that cases with rapid progression are more likely to be promptly identified and diagnosed, consistent with the observations by Andreasen et al. (Andreasen et al. 2013).

##### 4.1.1.4 Initial Systolic Blood Pressure

Each 1 mmHg increase in initial systolic blood pressure post‐onset was associated with a 0.7% increase in the risk of high hemorrhage volume (OR = 1.007). This aligns with the findings of Ohkuma et al., further emphasizing the crucial role of early blood pressure management (Ohkuma et al. 2025). However, it is important to note that this association should not be interpreted as suggesting that a small increase in blood pressure from very low values (e.g., 80 mmHg) would have the same clinical impact as an increase from hypertensive levels. The effect of blood pressure on hemorrhage volume may vary depending on the baseline level of pressure, with higher blood pressure values likely contributing more significantly to hemorrhage severity.

##### 4.1.1.5 Saccular Aneurysm Morphology

Saccular aneurysms significantly increased the risk of high hemorrhage volume (OR = 9.005) compared to other morphologies. This may be related to the hemodynamic characteristics of saccular aneurysms, as suggested by Shiba et al.’s computational fluid dynamics study (Shiba et al. 2017). Cerebral aneurysm morphology can potentially impact the hemorrhage volume in subarachnoid hemorrhage (SAH) through its influence on hemodynamics. Studies using computational fluid dynamics (CFD) analysis have shown that differences in aneurysm shape and geometry affect blood flow patterns and hemodynamic trends within the aneurysm (33). Concentrated high‐velocity inflow jets, complex multi‐vortex flows, and extremely high wall shear stress (WSS) have been correlated with aneurysm formation and progression (Zhai et al. 2022). Abnormal hemodynamics likely contributes to the weakening of the aneurysm wall and increase the risk of rupture. After rupture, the size and geometry of the aneurysm may determine the initial volume of bleeding into the subarachnoid space. Larger aneurysms with wide necks or irregular shapes may allow for greater initial hemorrhage volume compared to smaller, more regular aneurysms. However, direct evidence linking aneurysm morphology to measured SAH hemorrhage volumes is still limited. Further research is needed to fully elucidate the relationship between aneurysm geometry, hemodynamics, and the severity of bleeding in SAH.

Despite the valuable insights provided by this study, several limitations must be acknowledged. The retrospective design inherently introduces potential biases; however, we employed rigorous statistical analyses and included a large multicenter cohort to minimize these effects. While some confounding factors, such as medication use and timing of interventions, were not consistently available across all centers, we have acknowledged this limitation and emphasized the need for future prospective studies. Additionally, although our study identified independent risk factors for hemorrhage volume in aSAH, it did not aim to establish a formal risk stratification model. Future research should focus on developing predictive scoring systems to enhance clinical applicability. Moreover, while some risk factors, such as hypertension and diabetes, are well‐documented, our study provides additional insights by analyzing their collective impact in a large, diverse population. Further investigations, including mechanistic studies, are needed to elucidate the specific relationship between diabetes and hemorrhage volume, as well as the hemodynamic mechanisms linking aneurysm morphology to bleeding severity. Finally, the lack of long‐term follow‐up data limits our ability to assess the impact of these risk factors on patient outcomes over time. Future prospective multicenter studies are warranted to validate and quantify these associations, as well as to evaluate targeted interventions that may improve prognosis in aSAH patients.

Building on the findings of this study, future research should consider conducting prospective multicenter studies to validate and quantify the impact of these risk factors. Mechanistic studies are needed to explore the specific relationship between diabetes and hemorrhage volume in aSAH. Developing and validating predictive models based on these risk factors would enhance risk assessment accuracy. Additionally, investigating the effects of targeted interventions on these factors could improve the prognosis of aSAH. Finally, exploring the hemodynamic mechanisms linking aneurysm morphology to hemorrhage volume will provide further insights.

## 5 Conclusion

This multicenter retrospective study identifies key risk factors for hemorrhage volume in aneurysmal subarachnoid hemorrhage (aSAH), including diabetes, hypertension, saccular aneurysm morphology, aneurysm size, and systolic blood pressure after onset. These factors are crucial for assessing patient risk and guiding management strategies. Early recognition and management of these factors, such as controlling systolic blood pressure, managing diabetes, and monitoring aneurysm characteristics, may reduce hemorrhage volume and improve outcomes. While age and time to first CT scan were associated with hemorrhage volume in univariate analysis, they were not independently significant. Further prospective studies are needed to validate these findings and explore other variables that may influence hemorrhage volume. These risk factors can help identify high‐risk patients and improve personalized treatment plans, ultimately aiming to reduce the impact of aSAH.

## Author Contributions

**Chenglong Li**: conceptualization, methodology, writing–original draft. **Yuan Wang**: methodology, software. **Tangming Peng**: data curation, investigation. **Jiangnan Wu**: validation, formal analysis. **Hongyu Wang**: supervision, visualization. **Jian Song**: project administration, resources. **Di Zhao**: writing–review and editing, conceptualization, data curation, funding acquisition. **Guang Feng**: writing–review and editing, supervision. **Lei Chen**: project administration, resources.

## Ethics Statement

This study was approved by the Ethics Committee of The Fourth Hospital of Hebei Medical University(2024‐SJ‐ky358) and consent to participate was waived because this study does not involve any human participants.

## Consent

The authors have nothing to report

## Conflicts of Interest

The authors declare that they have no competing interests.

### Peer Review

The peer review history for this article is available at https://publons.com/publon/10.1002/brb3.70498

## Data Availability

Data is provided within the manuscript files.
